# Automated counting for *Plasmodium falciparum *cytoadherence experiments

**DOI:** 10.1186/1475-2875-10-91

**Published:** 2011-04-16

**Authors:** Douglas Paton, Brian Faragher, Khairul MF Mustaffa, Tadge Szestak, Steve D Barrett, Alister G Craig

**Affiliations:** 1 Liverpool School of Tropical Medicine, Pembroke Place, Liverpool L3 5QA, UK; 2 Department of Physics, University of Liverpool, Liverpool L69 3BX, UK; 3 Institute for Research in Molecular Medicine (INFORMM), Health Campus, Universiti Sains Malaysia, Kubang Kerian, 16150 Kelantan, Malaysia; 4 Research Institute for Science and Technology in Medicine, Keele University, Keele, Staffordshire ST5 5BG, UK

## Abstract

**Background:**

The ability of mature forms of *Plasmodium falciparum *infected erythrocytes to bind to a range of host receptors including those displayed on endothelial cells has been associated with the pathology of this infection. Investigations into this adhesive phenomenon have used protein and cell-based adhesion assays to quantify the ability of infected red blood cells to bind. These adhesion assays tend to have relatively high inherent variability and so require multiple experiments in order to provide good quantitation. This means that investigators doing these experiments must count many fields of adherent parasites, a task that is time-consuming and laborious. To address this issue and to facilitate cytoadherence research, developed automated protocols were developed for counting parasite adhesion.

**Methods:**

Parasite adhesion assays were mainly carried out under static conditions using purified receptors, which is the simplest form of these assays and is translatable to the field. Two different software platforms were used, one commercial (Image Pro-Plus (Media Cybernetics)) and one available in the public domain (ImageSXM) based on the freely available NIH Image software. The adhesion assays were performed and parasite binding quantified using standard manual techniques. Images were also captured using video microscopy and analysed using the two automated systems. The results generated by each system were compared using the Bland and Altman method for assessing the agreement between two methods.

**Results:**

Both automated counting programs showed concordance compared to the 'gold standard' manual counting within the normal range of adhesion seen with these assays, although the ImageSXM technique had some systematic bias. There was some fall-off in accuracy at very high parasite densities, but this can be resolved through good design of the experiments. Cell based assays were also used as inputs to one of the automated systems (ImageSXM) and produced variable, but encouraging, results.

**Conclusions:**

The automated counting programs are an accurate and practical way of quantifying static parasite binding assays to purified proteins. They are less accurate when applied to cell based systems, but can still provide a reasonable level of accuracy to give a semi-quantitative readout.

## Background

*Plasmodium falciparum *malaria remains a major burden in terms of its contribution to morbidity and mortality in resource poor countries [1]. Despite many years of research the basis of severe disease is still unclear, although clearly relates to be pro-inflammatory nature of the infection. The ability of the parasite infected red blood cell (pRBC) to bind to host receptors has been associated with pathology [2], with several studies attempting to correlate specific binding phenotypes with clinical outcome [3-6]. The basis of these experiments is often the use of static parasite binding assays, either using purified receptors or cells, either expressing specific receptors or host-derived endothelium. An inherent factor of these binding assays is their variability, requiring the enumeration of many fields and replicate experiments to produce accurate values for parasite binding. Counting parasites manually is a tedious and time-consuming task and so investigators will tend to minimize the amount of fields being counted, as well as having a small but significant element of observer bias. To address these issues we decided to develop automated counting systems, particularly for static assays with purified receptors, and validate them against the 'gold standard' of manual counting. The use of automated systems for counting cell-based assays was also considered. These assays have the additional difficulty in terms of automating counting due to the high 'background' of particulate matter caused by the cellular material comprising the target.

## Methods

### Parasite binding assays

#### Parasite culture

The parasite line ItG-ICAM [7] was used throughout. Parasites were cultured in RPMI 1640 medium (supplemented with 37.5 mM HEPES, 7 mM D-glucose, 6 mM NaOH, 25 μg ml^-1 ^gentamicin sulphate, 2 mM L-glutamine and 10% human serum) at a pH of 7.2, in a gas mixture of 96% nitrogen, 3% carbon dioxide and 1% oxygen. Parasites were synchronized using 5% sorbitol. Prior to use, parasites were washed twice in binding buffer (RPMI 1640 medium, supplemented with 6 mM glucose, pH 7.2) and re-suspended at 1% haematocrit (Coulter counter) and 3% parasitaemia (Giemsa staining). All parasite lines are subject to antigenic switching *in vitro *and so stabilates of ICAM-1 selected ItG-ICAM were used up to three weeks post-selection to minimize the effect of mixed populations.

#### Endothelial cells

Characterized human umbilical vein endothelial cells (HUVEC) and human dermal microvascular endothelial cells (HDMEC) were obtained from Promocell. Cells were grown in manufacturer's recommended medium and then frozen down in 7.5% DMSO/92.5% FCS. HUVEC and HDMEC were used at 6^th ^passage.

### Static protein assay

Static protein binding assays were carried out as previously described [8]. Briefly, 2 μl of ICAM-1-Fc (25, 50 or 100 μg ml^-1^) [9] or CD36 (25 μg ml^-1^) (R&D Systems) were spotted onto 60 mm diameter bacteriological Petri dishes and incubated in a humidified chamber for 2 h at 37°C. Proteins were aspirated off and dishes blocked overnight at 4°C in 1% BSA/PBS. Blocking solution was removed, the dish washed in binding buffer and 2 ml of parasite suspension at 3% parasitaemia and 1% haematocrit added to each dish. Dishes were incubated at 37°C for 1 h, with re-suspension for every 10 min. Unbound RBC and pRBC were removed by repeated washing, bound cells fixed with 1% glutaraldehyde for 1 h and then stained with 5% Giemsa for 20 min. Levels of ICAM-1 adhesion were quantitated microscopically or using automated image analysis (see below). CD36 adhesion was included solely as a positive control for the binding assay and was not included in the automated counting analysis in this study.

### Static cell assay

Static cell binding assays were carried out using a modified version of a previously described method [8]. HUVEC or HDMEC (6th passage) were seeded onto 1% gelatin-coated 13 mm Thermanox coverslips (Nalgene, Nunc). Once confluent, they were incubated overnight at 37°C with or without 0.5 ng ml^-1^rTNF (Biosource International). Cells were washed with binding buffer and incubated with 0.5 ml of parasite suspension (3% parasitaemia, 1% haematocrit) for 1 h at 37°C, with gentle resuspension every 10 min. A 1 h gravity wash removed unbound cells, adherent cells were fixed using 2% glutaraldehyde for 1 h and then stained with 5% Giemsa for 20 min. Coverslips were dried and mounted on slides using DPX mounting buffer (BDH Lab Supplies). Levels of adhesion were quantitated microscopically or using automated image analysis (see below).

### Image capture

Microscopy and image capture was carried out using a Nikon Eclipse TE2000 microscope with cooled CCD camera (QICAM) and remotely operated stage (Prior Optiscan) using the Image-Pro Plus 5.1 suite of image capture tools (IPP 5.1, Media Cybernetics) at 300× magnification. Eleven separate replicate experiments were carried out with twenty fields captured per assay.

pRBC were manually counted at 200× magnification. 6 × 0.25 mm^2 ^fields were selected by the user and counted for each of 12 ICAM-1 protein spots (3 dilutions plus negative control, in triplicate) per plate (CD36 was used only as a control for binding), and the mean of these counts multiplied by 4 to give a count of pRBC/mm^2 ^(4 × 0.25 mm^2 ^fields).

### Image analysis

#### Image Pro Plus

As an adjunct to image capture using IPP 5.1, a program was written using the Visual Basic for Applications (Microsoft Corporation) programming language that allowed any specified number of images to be analysed *simultaneously *within IPP 5.1. The program was then able to export the cell count information and user defined attributes to an Excel (Microsoft Corporation) database, which had been pre-prepared to analyse the data and output an pRBC/mm^2 ^result.

Following image capture, adherent pRBC appear as dark spots on a light background. Therefore IPP 5.1 was calibrated to recognize dark areas on the image. After initial testing, 3 potential sources of error were identified: incorrectly including uninfected red blood cells (uRBC) and non-cellular objects such as dust and dark marks on the camera lens in the final count, and incorrectly counting several clustered or overlapping cells as one single dark object. These problems were addressed as follows:

IPP 5.1 includes an algorithm which resolves clustering by determining the mean area of objects it identifies as single (assuming, as in this case, that area was normally distributed about the mean) and then dividing the area of any objects identified as clusters (anything beyond 2 standard deviations from the mean 'single object' area) by the mean single object area and adjusting the count accordingly.

The area of objects such as dust, fibres and marks on the camera lens were measured using the IPP 5.1 image analysis suite. These were then compared to the area of pRBC.

Un-infected cells: Uninfected RBC are generally un- or lightly stained when compared to pRBC and appear as brighter objects in images. Also, as a result of them maintaining their concave shape and in contrast to pRBC, they have a tendency to be brighter in the centre. With this in mind, attributes based on optical density, the degree of 'clumpiness' (patchiness in the optical density throughout the cell) and the 'margination' (the difference in optical density between the centre and the border of the cell) were measured and compared with those of pRBC.

#### ImageSXM

*Image SXM *[10] is an image analysis application that originated as a spin-off from the public domain software *NIH Image *[11] in the 1990s (developed at the U.S. National Institutes of Health). The software is continuously being updated and extended to handle images from various types of scanning microscopes (~50 image file formats) and image processing and analysis routines are being added to help research scientists working in the physical sciences (especially nanoscience), earth sciences and life sciences. Although many routines were developed with scanning microscopy in mind, a substantial part of the software is equally applicable to images from light microscopes. Using *Image SXM *as a starting platform, specialist image analysis solutions have been developed that address the needs of users in the life sciences for specific applications. *MIASMA *(Microscopy Image Analysis Software for Medical Applications) [12] is the result of a number of these specialist applications, such as the Parasite Counting Analysis (PCA) described here, having some common ground and so benefiting from being considered as part of a larger, overarching project.

The PCA routines were written in the Pascal source code of *Image SXM *using the CodeWarrior compiler system (Metrowerks). The PCA takes minimal input from the user (specifying limits on the minimum and maximum sizes of objects that should be analysed as potential candidates for pRBC) and then carries out a batch analysis of all images in a set of folders. The output is (i) a text file giving the details of the pRBC count in each image and (ii) a summary of the batch analysis with the average pRBC count for images in each folder. In addition, a colour-coded image is generated for each image that shows the pRBC that were identified and counted. After the PCA routines have completed, a blink comparator allows the user to compare these output images with the original images to determine whether or not the analysis ran with satisfactory accuracy.

Each image is processed to compensate for differences in brightness and/or contrast and then a background subtraction routine is applied to minimize the influence of cell structure on pRBC recognition. The geometric characteristics of each candidate for a pRBC, which appear as dark objects on a light background, are measured -- area, perimeter, and lengths of the major and minor axes of the best-fit ellipse. These measurements are used to (i) veto those objects that do not meet geometric criteria and (ii) determine if the object is two or more touching pRBC.

The criteria for valid pRBC were determined from a batch of test images:

(i) perimeter^2^/area < 100 (a test of the convolution of the object's outline)

(ii) major axis/minor axis < 3 (a test of the aspect ratio of the object)

The criteria for an object being recognized as *n *touching pRBC use a combination of area, perimeter and aspect ratio, with the value of *n *being calculated from the object's area relative to the mean area of all objects found in the image.

The blink comparator can be used to check an image with the corresponding output image that shows which pixels were identified as cytoplasm and pRBC. This can be done on a selection of images before or after a full PCA run is executed. If there is any consistent under/overestimate of the pRBC count then the user can adjust global parameters that fine tune the PCA algorithms and run the analysis again. If there is no consistent under/over-estimate of the pRBC count but some objects in some images have been identified incorrectly, then the user can opt to apply a manual override to adjust the analysis on an image-by-image basis.

### Statistical analysis

The results obtained using the Image ProPlus and SXM automatic methods (Additional file 1) were compared against the manual method using the Bland and Altman method [13] for assessing the agreement between two measurements. The arithmetic means of the triplicate readings taken on each of 11 plates at each of the four concentrations were evaluated. The differences between the methods followed approximate Normal distributions at each of the four concentrations tested, although the variation in these differences increased with concentration level. Levels of agreement are described graphically using a Bland and Altman plot (differences between the automatic and manual measurements versus the averages of the two measurements) for all four concentrations combined. Mean differences (automatic minus manual) with their 95% reference ranges (mean difference ± 2*standard deviation) are reported for each concentration separately. The 95% reference range represents the interval within which 95% of differences between the two methods are likely to fall.

## Results

**Figure 1:**
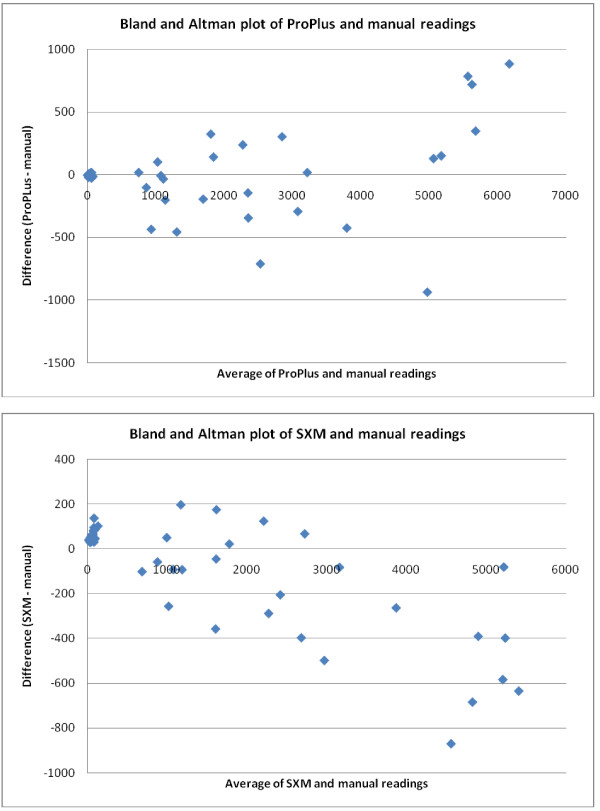
**Comparison of binding results**. Bland and Altman plots comparing Image Pro Plus (ProPlus) and ImageSXM (SXM) automated counting techniques with manual counts for adhesion assays on plastic to ICAM-1 using the *P. falciparum *line ItG-ICAM.

The Bland and Altman plots (Figure 1) show clearly that the variation in the differences between the automatic and manual measurements tended to increase as the (average) count level increased (and hence as concentration increased) - and this was particularly marked for the SXM method.

For the comparison of the Image ProPlus and manual methods, the mean difference between the two measurements was not statistically significant for any of the individual concentration levels, indicating an absence of any systematic bias between these two methods. However, the Bland and Altman plot does suggest a tendency for the automatic Image ProPlus method to systematically produce higher counts than the manual method when the counts are very large (> 6,000).

For the comparison of the SXM and manual methods, the mean difference between the two measurements was statistically significant for the 0 μg/ml and 50 μg/ml concentration levels and was close to statistical significance (0.05 < p < 0.10) for the 12.5 μg/ml and 25 μg/ml concentration levels. The automatic SXM method tended to systematically over-estimate counts at the 0 μg/ml and 12.5 μg/ml concentration levels but to under-estimate counts at the 25 μg/ml and 50 μg/ml concentration levels.

Table 1 shows the 95% reference range for the differences between the two methods at each of the four concentrations tested. Proportionally, the reference ranges represent, for both automatic methods relative to the corresponding manual counts, potential differences between the counting methods of less than (approximately) ± 40% for the 12.5 μg/ml concentration and ± 30% for the 25 μg/ml and 50 μg/ml concentrations.

**Table 1:** Mean (standard deviation) counts and differences for manual and automatic counting methods

ICAM-1 Concentration	Method			ProPlus - Manual		SXM - Manual	
	Manual	ProPlus	SXM	difference	95% reference range	difference	95% reference range
0	27 (14)	27 (20)	92 (40)	0 (11)	-22 to 22 [-81% to 81%]	65 (32)	2 to 128 [7% to 474%]
12.5	495 (634)	473 (641)	538 (618)	-21 (83)	-186 to 144 [-38% to 29%]	43 (77)	-110 to 197 [-22% to 40%]
25	1723 (701)	1574 (646)	1596 (645)	-150 (318)	-786 to 487 [-46% to 28%]	-127 (192)	-510 to 257 [-30% to 15%]
50	4577 (1086)	4730 (1349)	4176 (946)	152 (553)	-954 to 1258 [-21% to 27%]	-401 (289)	-979 to 177 [-21% to 4%]

Additional file 2 shows data for a single experiment using ImageSXM to analyse endothelial cell binding assays compared to manual counts.

## Discussion

One of the major challenges for malaria cytoadherence assays has been the counting. These assays are also notoriously variable, requiring several replicates that add to this burden. In this paper we have investigated two automated methods for parasite counting based on the simplest format, namely static assays performed with purified protein. The counting software used represent commercial (Image ProPlus) and publically-available (ImageSXM) platforms and we have attempted to optimize both so that they are able to cover a broad range of values generated by the assays. Of the two methods the Image ProPlus platform more closely represented the counts obtain manually with no significant differences at all concentrations tested. ImageSXM did show some bias, and this varied based on the concentration of receptor used, and therefore the number of adherent parasites per field.

The superiority of the commercial software may reflect, in part, the better user interface available with the Image ProPlus package and better familiarity with the Image SXM software might have improved the outcome. However, despite having access to an expert on ImageSXM (SBD), the software backage was tested without extensive intervention to see how it would perform under 'standard' conditions. As well as the small bias seen with ImageSXM we also encountered some problems with the analysis of images from different batches, where SXM had less flexibility to cope with changes in exposure and contrast. Despite this SXM still performed well producing results that were consistent with the other methods and without the need to purchase commercial software. ImageSXM software was also applied to cell-based assays, which bring an added level of complexity to automated counting due to the presence of many 'particles' contributed by the endothelium. The results (Additional file 2) are still too variable to be confident in replacing manual counting for this assay format but they were at the very least encouraging and with the need to move to more 'biologically-relevant' adhesion assays using primary endothelial cells this could become an important issue. As stated in the Results section, the Bland and Altman plot suggested a tendency for the automatic Image ProPlus method to systematically produce higher counts than the manual method when the counts are very large (> 6,000) but as only a few very large observations were obtained, this finding requires further investigation to identify whether this is a real effect or not.

In conclusion, an automated counting procedure has been successfully applied to malaria parasite adhesion assays that is able to produce accurate results over a range of values, with some loss of accuracy only at very high levels of binding. A related, freely-available package (ImageSXM) produced some systematic errors when compared to the 'gold-standard' manual counting but still provided data that were consistent with the other techniques, and with the flexibility to be applied to the next generation of cell-based adhesion assays.

## Competing interests

The authors declare that they have no competing interests.

## Authors' contributions

DP carried out the binding assays, designed the IPP counting routine, performed the counting (manual and automatic), participated in the design of the study and drafted the section on IPP counting. KM and TS performed binding assays and performed the counting. SB wrote the routines for MIASMA based on ImageSXM and drafted the section on MIASMA-based counting. BF performed the statistical analysis and drafted the section on this analysis. AC conceived of and designed the study and drafted the manuscript. All authors read and approved the final manuscript.

## Supplementary Material

Additional file 1 **Table of pRBC binding data**. The binding results using manual and automated counting for replicate experiments to measure pRBC adhesion to ICAM-1. Click here for file

Additional file 2 **Automated counting of cell-based assays**. An example of cell-based adhesion assay automated counting using Image SXM from a single assay. The results from manual and SXM methods have been plotted against each other to demonstrate the level of concordance, and three images have been included to provide one example of successful automated counting and two images where the automated system does not match the manual counts. Click here for file

## References

[B1] Hay SI Okiro EA Gething PW Patil AP Tatem AJ Guerra CA Snow RW Estimating the global clinical burden of *Plasmodium falciparum *malaria in 2007 PLoS Med 2010 7 e1000290 10.1371/journal.pmed.1000290 20563310 PMC2885984

[B2] Rowe JA Claessens A Corrigan RA Arman M Adhesion of *Plasmodium falciparum*-infected erythrocytes to human cells: molecular mechanisms and therapeutic implications Expert Rev Mol Med 2009 11 e16 10.1017/S1462399409001082 19467172 PMC2878476

[B3] Udomsangpetch R Reinhardt PH Schollaardt T Elliott JF Kubes P Ho M Promiscuity of clinical *Plasmodium falciparum *isolates for multiple adhesion molecules under flow conditions J Immunol 1997 158 4358 4364 9126999

[B4] Newbold C Warn P Black G Berendt A Craig A Snow B Msobo M Peshu N Marsh K Receptor-specific adhesion and clinical disease in *Plasmodium falciparum* Am J Trop Med Hyg 1997 57 389 398 9347951 10.4269/ajtmh.1997.57.389

[B5] Ho M Schollaardt T Niu X Looareesuwan S Patel KD Kubes P Characterization of *Plasmodium falciparum*-infected erythrocyte and P-selectin interaction under flow conditions Blood 1998 91 4803 4809 9616180

[B6] Rogerson SJ Tembenu R Dobano C Plitt S Taylor TE Molyneux ME Cytoadherence characteristics of *Plasmodium falciparum*-infected erythrocytes from Malawian children with severe and uncomplicated malaria Am J Trop Med Hyg 1999 61 467 472 10497992 10.4269/ajtmh.1999.61.467

[B7] Ockenhouse CF Betageri R Springer TA Staunton DE *Plasmodium falciparum*-infected erythrocytes bind ICAM-1 at a site distinct from LFA-1, Mac-1, and human rhinovirus Cell 1992 68 63 69 10.1016/0092-8674(92)90206-R 1346257

[B8] Gray C McCormick C Turner G Craig A ICAM-1 can play a major role in mediating *P. falciparum *adhesion to endothelium under flow Mol Biochem Parasitol 2003 128 187 193 10.1016/S0166-6851(03)00075-6 12742585

[B9] Craig AG Pinches R Khan S Roberts DJ Turner GD Newbold CI Berendt AR Failure to block adhesion of *Plasmodium falciparum*-infected erythrocytes to ICAM-1 with soluble ICAM-1 Infect Immun 1997 65 4580 4585 9353036 10.1128/iai.65.11.4580-4585.1997 PMC175657

[B10] ImageSXM http://www.liv.ac.uk/~sdb/ImageSXM/(Date accessed: 25/02/11)

[B11] NIH Image http://rsb.info.nih.gov/nih-image/(Date accessed: 25/02/11)

[B12] MIASMA http://www.liv.ac.uk/~sdb/MIASMA/PDFs/MIASMA-PCA-v5.pdf (Date accessed: 25/02/11)

[B13] Bland JM Altman DG Statistical methods for assessing agreement between two methods of clinical measurement Lancet 1986 i 307 310 10.1016/S0140-6736(86)90837-8 2868172

