# Supplementary material for: Automated counting for Plasmodium falciparum cytoadherence experiments
Source: Malar J. 2011 Apr 16;10:91. doi: 10.1186/1475-2875-10-91 (PMC3094228; doi:10.1186/1475-2875-10-91)
Supplement: Additional file 1 — Table of pRBC binding data. The binding results using manual and automated counting for replicate experiments to measure pRBC adhesion to ICAM-1. [file 1475-2875-10-91-S1.DOCX]

|  | Supplementary Table 1 – Counting data |  |  |  |  |  |  |  |  |  |  |  |  |
| --- | --- | --- | --- | --- | --- | --- | --- | --- | --- | --- | --- | --- | --- |
|  |  |  |  |  |  |  |  |  |  |  |  |  |  |
|  |  |  |  |  |  |  |  |  |  |  |  |  |  |
|  |  |  |  |  |  |  |  |  |  |  |  |  |  |
|  |  |  |  |  |  |  |  |  |  |  |  |  |  |
|  |  |  | Manual |  |  |  | Image Pro-Plus v5.1 | |  |  | ImageSXM | |  |
|  | ICAM-1 coating concentration (ug/ ml) | Expt. No. |  |  |  |  |  |  |  |  |  |  |  |
|  |  |  | Mean per mm2 | | SE |  | Mean per mm2 | | SE |  | Mean per mm2 | | SE |
|  | 0 | 1 | 41.1 |  | 24.3 |  | 42.7 |  | 18.7 |  | 101.3 |  | 27.8 |
|  | 12.5 |  | 74.0 |  | 31.1 |  | 46.7 |  | 15.4 |  | 104.0 |  | 20.1 |
|  | 25 |  | 1642.7 |  | 650.7 |  | 1966.7 |  | 289.2 |  | 1597.3 |  | 175.7 |
|  | 50 |  | 5265.3 |  | 2115.9 |  | 5985.3 |  | 1022.0 |  | 5184.0 |  | 990.9 |
|  |  |  |  |  |  |  |  |  |  |  |  |  |  |
|  | 0 | 2 | 44.4 |  | 23.2 |  | 29.3 |  | 8.1 |  | 126.7 |  | 6.6 |
|  | 12.5 |  | 79.8 |  | 16.1 |  | 68.0 |  | 12.2 |  | 125.3 |  | 18.0 |
|  | 25 |  | 1791.3 |  | 372.1 |  | 1596.0 |  | 562.5 |  | 1434.0 |  | 453.8 |
|  | 50 |  | 5729.3 |  | 679.0 |  | 6613.3 |  | 940.4 |  | 5095.3 |  | 557.5 |
|  |  |  |  |  |  |  |  |  |  |  |  |  |  |
|  | 0 | 3 | 40.7 |  | 21.3 |  | 52.0 |  | 22.8 |  | 135.3 |  | 16.6 |
|  | 12.5 |  | 87.3 |  | 13.7 |  | 73.3 |  | 13.9 |  | 188.7 |  | 20.2 |
|  | 25 |  | 976.2 |  | 647.0 |  | 1077.3 |  | 339.1 |  | 1026.0 |  | 405.3 |
|  | 50 |  | 4998.2 |  | 1761.9 |  | 5126.7 |  | 1185.5 |  | 4129.3 |  | 923.7 |
|  |  |  |  |  |  |  |  |  |  |  |  |  |  |
|  | 0 | 4 | 18.0 |  | 8.4 |  | 24.0 |  | 6.1 |  | 69.3 |  | 5.5 |
|  | 12.5 |  | 40.7 |  | 6.1 |  | 40.0 |  | 2.3 |  | 73.3 |  | 8.8 |
|  | 25 |  | 741.8 |  | 97.4 |  | 758.7 |  | 149.2 |  | 640.0 |  | 69.0 |
|  | 50 |  | 5504.0 |  | 173.5 |  | 5852.0 |  | 192.9 |  | 4921.0 |  | 2.5 |
|  |  |  |  |  |  |  |  |  |  |  |  |  |  |
|  | 0 | 5 | 42.0 |  | 24.3 |  | 57.3 |  | 17.5 |  | 121.3 |  | 39.9 |
|  | 12.5 |  | 58.9 |  | 26.3 |  | 44.0 |  | 8.3 |  | 144.7 |  | 45.7 |
|  | 25 |  | 1152.2 |  | 952.9 |  | 716.0 |  | 340.4 |  | 896.0 |  | 354.3 |
|  | 50 |  | 5099.6 |  | 1422.7 |  | 5250.0 |  | 773.3 |  | 4709.3 |  | 595.3 |
|  |  |  |  |  |  |  |  |  |  |  |  |  |  |
|  | 0 | 6 | 21.1 |  | 16.9 |  | 28.0 |  | 6.1 |  | 157.3 |  | 14.5 |
|  | 12.5 |  | 41.8 |  | 4.2 |  | 32.0 |  | 6.1 |  | 108.0 |  | 14.0 |
|  | 25 |  | 1536.2 |  | 1005.9 |  | 1078.7 |  | 344.4 |  | 1710.7 |  | 568.3 |
|  | 50 |  | 5441.8 |  | 1034.5 |  | 4504.0 |  | 688.9 |  | 5044.0 |  | 587.8 |
|  |  |  |  |  |  |  |  |  |  |  |  |  |  |
|  | 0 | 7 | 32.9 |  | 11.2 |  | 38.7 |  | 2.7 |  | 96.7 |  | 8.7 |
|  | 12.5 |  | 52.7 |  | 9.5 |  | 69.3 |  | 17.4 |  | 99.3 |  | 5.7 |
|  | 25 |  | 1127.8 |  | 876.4 |  | 1093.3 |  | 532.3 |  | 1035.3 |  | 436.4 |
|  | 50 |  | 5174.0 |  | 2629.8 |  | 5958.7 |  | 1591.5 |  | 4490.7 |  | 1256.7 |
|  |  |  |  |  |  |  |  |  |  |  |  |  |  |
|  | 0 | 8 | 4.0 |  | 3.1 |  | 1.3 |  | 1.3 |  | 44.0 |  | 9.9 |
|  | 12.5 |  | 1239.8 |  | 324.4 |  | 1037.3 |  | 401.4 |  | 1146.7 |  | 245.2 |
|  | 25 |  | 2526.7 |  | 449.6 |  | 2181.3 |  | 304.0 |  | 2321.3 |  | 264.8 |
|  | 50 |  | 3224.4 |  | 538.0 |  | 2930.7 |  | 256.2 |  | 2726.7 |  | 310.1 |
|  |  |  |  |  |  |  |  |  |  |  |  |  |  |
|  | 0 | 9 | 26.4 |  | 15.3 |  | 5.3 |  | 2.7 |  | 54.7 |  | 8.7 |
|  | 12.5 |  | 1773.8 |  | 595.4 |  | 1914.7 |  | 460.6 |  | 1795.3 |  | 305.4 |
|  | 25 |  | 2885.3 |  | 819.8 |  | 2173.3 |  | 497.3 |  | 2488.7 |  | 378.2 |
|  | 50 |  | 4007.3 |  | 1006.0 |  | 3581.3 |  | 468.8 |  | 3744.0 |  | 498.7 |
|  |  |  |  |  |  |  |  |  |  |  |  |  |  |
|  | 0 | 10 | 10.0 |  | 1.8 |  | 4.0 |  | 4.0 |  | 45.3 |  | 1.8 |
|  | 12.5 |  | 1078.4 |  | 606.9 |  | 1070.7 |  | 343.4 |  | 1274.7 |  | 496.3 |
|  | 25 |  | 2421.6 |  | 848.4 |  | 2276.0 |  | 540.4 |  | 2133.3 |  | 524.1 |
|  | 50 |  | 3207.3 |  | 539.8 |  | 3224.0 |  | 392.9 |  | 3124.7 |  | 174.9 |
|  |  |  |  |  |  |  |  |  |  |  |  |  |  |
|  | 0 | 11 | 14.2 |  | 13.5 |  | 9.3 |  | 4.8 |  | 58.7 |  | 11.6 |
|  | 12.5 |  | 913.3 |  | 464.9 |  | 810.7 |  | 382.2 |  | 854.7 |  | 293.4 |
|  | 25 |  | 2154.0 |  | 650.8 |  | 2392.0 |  | 644.9 |  | 2277.3 |  | 467.9 |
|  | 50 |  | 2696.0 |  | 109.7 |  | 2998.7 |  | 218.8 |  | 2762.7 |  | 153.5 |
