# Supplementary figures and images for: Automated counting for Plasmodium falciparum cytoadherence experiments
Source: Malar J. 2011 Apr 16;10:91. doi: 10.1186/1475-2875-10-91 (PMC3094228; doi:10.1186/1475-2875-10-91)

|  |  |  |  |  |  |  |  |  |  |  |  |  |  |
| --- | --- | --- | --- | --- | --- | --- | --- | --- | --- | --- | --- | --- | --- |
| Supplementary Fig 1 |  |  |  |  |  |  |  |  |  |  |  |  |  |
| |  | | --- | |  |  |  |  |  | Cell-based assay | |  |  |  |  |  |  |
| 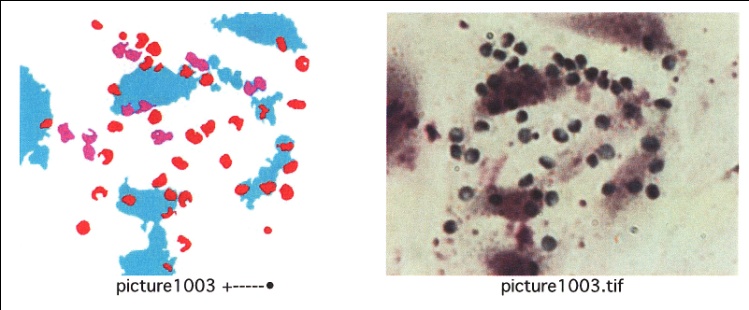 |  |  |  |  |  |  |  |  |  |  |  |  |  |
|  |  |  |  |  |  | ImageSXM | Manual |  | | | | | |
|  |  |  |  |  |  | 25 | 21 |
|  |  |  |  |  |  | 33 | 22 |
|  |  |  |  |  |  | 47 | 48 |
|  |  |  |  |  |  | 27 | 25 |
|  |  |  |  |  |  | 51 | 42 |
|  |  |  |  |  |  | 13 | 8 |
| 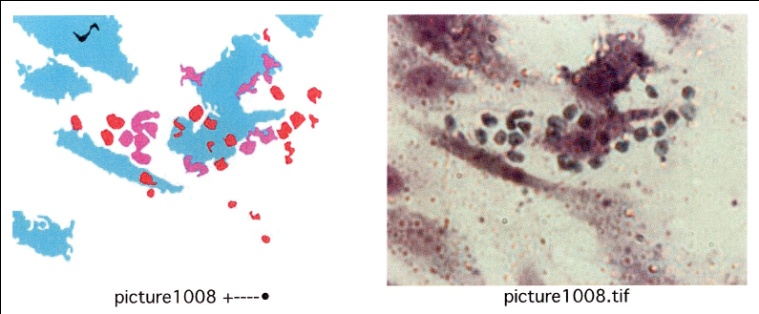   |  | | --- | |  |  |  |  |  | 29 | 25 |
|  |  |  |  |  |  | 37 | 26 |
|  |  |  |  |  |  | 29 | 31 |
|  |  |  |  |  |  | 28 | 30 |
|  |  |  |  |  |  | 20 | 16 |
|  |  |  |  |  |  | 25 | 29 |
|  |  |  |  |  |  | 23 | 20 |
|  |  |  |  |  |  | 43 | 29 |
|  |  |  |  |  |  | 55 | 32 |  |  |  |  |  |  |
|  |  |  |  |  |  | 46 | 46 |  |  |  |  |  |  |
|  |  |  |  |  |  | 26 | 18 |  |  |  |  |  |  |
|  |  |  |  |  |  | 26 | 21 |  |  |  |  |  |  |
| |  | | --- | |  |  |  |  |  | 28 | 23 |  |  |  |  |  |  |
| 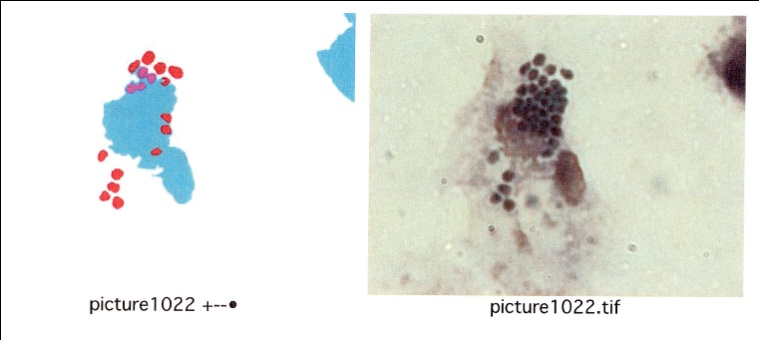 |  |  |  |  |  | 16 | 11 |  |  |  |  |  |  |
|  |  |  |  |  |  | 13 | 11 |  |  |  |  |  |  |
|  |  |  |  |  |  | 17 | 35 |  |  |  |  |  |  |
|  |  |  |  |  |  | 24 | 23 |  |  |  |  |  |  |
|  |  |  |  |  |  | 25 | 23 |  |  |  |  |  |  |
|  |  |  |  |  |  | 18 | 14 |  |  |  |  |  |  |
|  |  |  |  |  |  | 49 | 50 |  |  |  |  |  |  |
|  |  |  |  |  |  | 41 | 46 |  |  |  |  |  |  |
|  |  |  |  |  |  | 51 | 56 |  |  |  |  |  |  |
|  |  |  |  |  |  | 39 | 46 |  |  |  |  |  |  |
|  |  |  |  |  |  | 38 | 45 |  |  |  |  |  |  |

Supplement: Additional file 2 — Automated counting of cell-based assays. An example of cell-based adhesion assay automated counting using Image SXM from a single assay. The results from manual and SXM methods have been plotted against each other to demonstrate the level of concordance, and three images have been included to provide one example of successful automated counting and two images where the automated system does not match the manual counts. [file 1475-2875-10-91-S2.DOCX]
